# COL-3, a Chemically Modified Tetracycline, Inhibits Lipopolysaccharide-Induced Microglia Activation and Cytokine Expression in the Brain

**DOI:** 10.1371/journal.pone.0057827

**Published:** 2013-02-28

**Authors:** Rawan Abdulhameed Edan, Yunus A. Luqmani, Willias Masocha

**Affiliations:** 1 Department of Pharmaceutical Chemistry, Faculty of Pharmacy, Kuwait University, Safat, Kuwait; 2 Department of Pharmacology and Therapeutics, Faculty of Pharmacy, Kuwait University, Safat, Kuwait; Virginia Commonwealth University, United States of America

## Abstract

Microglia activation results in release of proinflammatory molecules including cytokines, which contribute to neuronal damage in the central nervous system (CNS) if not controlled. Tetracycline antibiotics such as minocycline inhibit microglial activation and cytokine expression during CNS inflammation. In the present study we found that administration of chemically modified tetracycline-3 (COL-3), inhibits lipopolysaccharide (LPS)-induced microglial and p38 MAPK activation, as well as the increase in TNF-α, but not IL-1β expression, in the brains of BALB/c mice. COL-3 has been described to have no antibacterial activity. We observed that COL-3 had no activity against a Gram-negative bacteria, *Escherichia coli*; however surprisingly, COL-3 had antibacterial activity against a Gram-positive bacteria *Staphylococcus aureus*, with a minimum inhibitory concentration of 1 mg/ml. Our data show that COL-3 has some antibacterial activity against *S. aureus*, inhibits LPS-induced neuroinflammation, and displays potential as a therapeutic agent for treatment of conditions involving CNS inflammation.

## Introduction

Neuroinflammation is an important factor in both the pathogenesis and progression of neurodegenerative diseases as well as in brain infections [1], [2], [3]. A vital component of neuroinflammation is the chronic activation of microglia which are the major inflammatory cells resident in the central nervous system (CNS) and which constitute its innate immune system. Activated microglia can release neurotrophic factors, pro-inflammatory cytokines such as tumor necrosis factor-alpha (TNF-α) and interleukin-1β (IL-1β), chemokines which attract inflammatory cells and many cytotoxic molecules including nitric oxide, oxygen radicals, eicosanoids and quinolic acid [4], [5]. Acute activation results in tissue repair and protective immune response induction. However, if it becomes chronic it can be deleterious to the brain, resulting in neurodegeneration. Such activation is observed during viral encephalitis, bacterial meningitis, multiple sclerosis, ischemia, trauma, Parkinson’s disease and similar conditions [1], [2], [3].

Drugs that inhibit microglia activation such as minocycline, a second-generation tetracycline antibiotic, have been reported to exert neuroprotective effects [6], [7], [8], [9], [10]. However, administration of minocycline over long periods of time may result in emergence of bacterial resistance to tetracycline antibiotics and also lead to undesirable side effects including disturbances of the commensal microflora. To overcome this problem, a series of chemically modified tetracyclines (CMTs) which have been attributed to lack anti-bacterial but retain anti-inflammatory activities have been synthesized. One example is CMT-3 { 6-dimethyl-6-deoxy-4-de(dimethylamino) tetracycline} also known as COL-3 [11], a potent inhibitor of matrix metalloproteases (MMPs) [11], [12] that has anti-tumor activities [13], [14], [15]. Being highly lipophilic COL-3 is expected to cross the blood brain barrier and therefore exert effects on cells within the brain [16], [17].

Lipopolysaccharide (LPS), an endotoxin from the outer membrane of gram-negative bacteria, is known to activate microglia [18], [19] and as such it is used frequently as a research agent for this purpose both *in vitro* and *in vivo* [20], [21], [22], [23]. Using LPS to activate microglia both *in vitro* and *in vivo* many research groups have shown that minocycline can inhibit microglia activation, and reduce the transcription and release of various cytokines and inflammatory molecules by microglia [24], [25], [26]. In the current study, we evaluated the effects of COL-3 on microglial activation and expression of pro-inflammatory cytokines in the brains of LPS inoculated BALB/c mice.

## Materials and Methods

### 2.1. Animals, LPS Administration and COL-3 Treatment

Female BALB/c mice (8 to 12 weeks old) were supplied by the breeding unit at the Health Sciences Center, Kuwait University. All animals were housed in temperature controlled (24±1°C) rooms with food and water available ad libitum. Efforts were made to minimize numbers and suffering of animals used. All procedures were approved by the Ethical Committee for the use of Laboratory Animals in Teaching and in Research Health Sciences Centre, Kuwait University.

Mice (n = 48) received a single intraperitoneal dose of LPS from *Escherichia coli* (strain O111:B4, Sigma-Aldrich, St Louis, MO, USA, n = 32) 1 mg/kg or its solvent (phosphate buffered saline; PBS, n = 16). Treatment with COL-3 (a gift from Galderma, Research and Development SNC, Les Templier, France) or its vehicle (1% methyl cellulose, MC), was by oral gavage administered at a volume of 12.5 µl/g body mass (equivalent to 40 mg/kg) commencing 2 days before LPS inoculation. LPS-inoculated drug control mice (n = 16) received MC daily, while the COL-3-treated mice (n = 16) received the drug once daily for 3 days. The choice of dose regimen was based on a previous report of effective minocyline reduction of LPS-induced microglia activation [22] and the dose of COL-3 that exerted antitumor activity [27]. Animals were periodically weighed and checked for signs of disease. On the appropriate day post inoculation and treatment they were anaesthetized with isoflurane and sacrificed by decapitation. Brains were removed and dissected at 24 h after LPS inoculation, snap frozen in liquid nitrogen and kept at −70°C prior to sectioning for immunohistochemistry or mRNA extraction.

### 2.2. Immunohistochemistry

Fresh-frozen, non-perfused brains at a level of the lateral ventricles and the septal nuclei were cut on a cryostat into 24 µm thick sections and thaw-mounted on chrome-alum gelatin–coated slides. Prior to immunohistochemical processing, the sections were fixed in methanol at −20°C for 10 min, left in a sterile cabinet for 10 min to evaporate methanol, and then rinsed in PBS. All sections were preincubated with 1% bovine serum albumin and 0.3% Triton X-100 in PBS (diluent used for all primary and secondary antisera) for 1 h at room temperature. Sections were incubated with the primary antibodies, rat monoclonal anti-CD11b antibody [M1/70] (1∶100, Abcam,Cambridge, UK) and rabbit anti-phospho-p38 mitogen-activated protein kinase (p38 MAPK) (Thr180/Tyr182) (1∶50, Cell Signaling Technology, Beverly, MA) in a humidified chamber at 4°C overnight. CD11b was used to identify LPS-induced activation of microglia similar to what has been described before [28], [29], [30], [31]. Sections were then rinsed in PBS and incubated with DyLight 594-conjugated Affinipure donkey Anti-rabbit IgG (H+L) (1∶100, Jackson ImmunoResearch Laboratories, West Grove, PA, USA) and Dylight 488 Affinipure Goat Anti-Rat IgG (H+L) (1∶100, Jackson ImmunoResearch) for 60 min. The sections were rinsed and mounted in ProLong® Gold antifade reagent (Invitrogen, USA). Images were taken using confocal microscopy with an Axio imager (Carl Zeiss MicroImaging GmbH, Germany).

### 2.3. Real Time RT-PCR

Gene transcripts of CD11b, interleukin (IL)-1β and tumor necrosis factor (TNF)-α were quantified in brains from COL-3-treated and MC-treated LPS-inoculated and uninoculated mice by real time PCR. Total RNA was extracted from one half of each of the fresh frozen brains, reverse-transcribed, and the transcripts levels quantified on an ABI Prism 7500 sequence detection system (Applied Biosystems) as described previously [23]. The sequences of the primers used are listed in Table 1. The Ct values for all cDNA samples were obtained using the ABI software. The amount of transcripts for each individual animal (n = 4 to 12 per group ) was normalized to cyclophilin. The relative amount of target gene transcripts was calculated using the 2^−ΔΔCt^ method as described previously [32]. These values were then used to calculate the mean and standard error of the relative expression of the target gene mRNA in the brain of un-inoculated and LPS-inoculated mice.

**Table 1:** PCR primer sequences of cyclophilin, CD11b and cytokines.

Gene	Polarity	Sequence 5′to 3′	GenBank*
Cyclophilin	Sense	GCTTTTCGCCGCTTGCT	X52803
	Anti-sense	CTCGTCATCGGCCGTGAT	
CD11b	Sense	TGCTTACCTGGGTTATGCTTCTG	NM-008401
	Anti-sense	CCGAGGTGCTCCTAAAACCA	
TNF-α	Sense	GGCTGCCCCGACTACGT	NM-013693
	Anti-sense	GACTTTCTCCTGGTATGAGATAGCAAA	
IL-1β	Sense	TGGTGTGTGACGTTCCCATT	NM-008361
	Anti-sense	CAGCACGAGGCTTTTTTGTTG	

* GenBank accession numbers.

### 2.4. Antibacterial Activity Evaluation

Briefly, the antibacterial activities of COL-3 was evaluated against a Gram-positive bacteria *Staphylococcus aureus* and a Gram-negative bacteria *Escherichia coli* using a disc diffusion method. Minimum inhibitory concentrations (MIC, mg/L) was determined for COL-3 against *S. aureus* only.

### 2.5. Statistical Analysis

Statistical analyses were performed using one way ANOVA followed by Newman-Keuls multiple comparison test using Graph Pad Prism software (version 5.0). Differences were considered significant when p<0.05. The results in the text and figures are expressed as mean ± S.E.M.

## Results

### 3.1. Effect of COL-3 on LPS-induced Microglia Activation in the Brain

**Figure 1:**
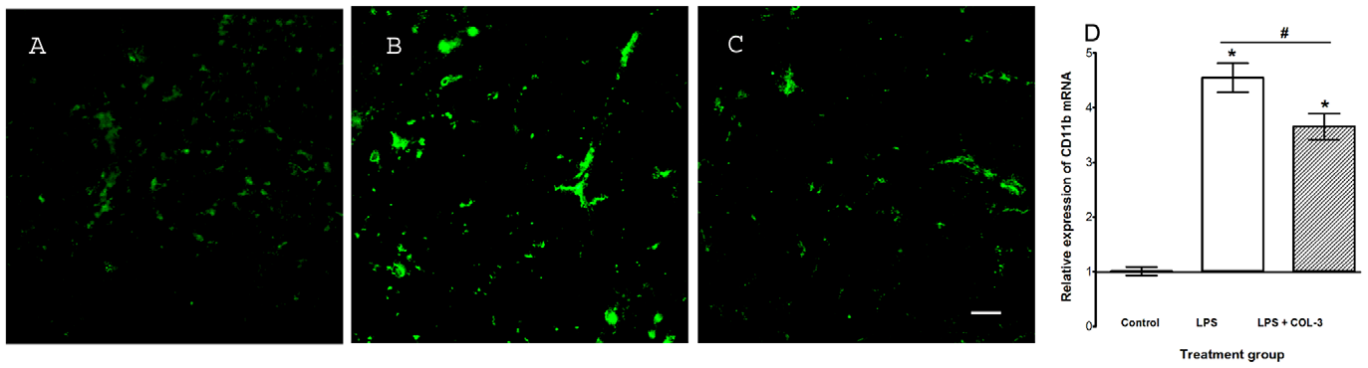
Effects of COL-3 on LPS-induced microglia activation in the brain. (A–C) Confocal laser microscopy images showing immunoreactivity of CD11b in the anterior commissure of fresh frozen sections of mice brains treated with vehicle (MC) (A), LPS (B) and LPS +40 mg/kg COL-3 (C). Immunoreactivity of CD11b was increased in LPS inoculated mice (B) compared to control mice (A) and was reduced by treatment with LPS+ COL-3 (C). Scale bar: 20 µm. (D) Relative expression of CD11b mRNA expression in brains of control mice, LPS-inoculated vehicle-treated and LPS-inoculated COL-3-treated mice at 24 h post inoculation. Each bar represents the mean ± S.E.M of the values obtained from four animals. *P<0.05 compared to control animals and #P<0.05 compared to LPS-inoculated mice pretreated with vehicle.

The effects of COL-3 on LPS-induced microglia activation was evaluated by both mRNA expression and immunohistochemical localization of target protein. Brain sections were immunostained for CD11b and also p38 MAPK; the latter because previous studies have indicated that minocycline blocks microglia activation via inhibition of p38 MAPK activation [33]. Confocal microscopy images were taken from the anterior commissure and the corpus callosum because strong immunoreactivity of p38 MAPK has been previously observed principally in these areas [34]. LPS-inoculated animals showed high reactivity of CD11b compared to vehicle-injected controls. Treatment with COL-3 reduced the LPS-induced increase in CD11b immunoreactivity (Fig. 1A–C). The levels of CD11b mRNA were also increased in the brains of LPS-inoculated mice compared to vehicle-injected controls. Treatment with COL-3 significantly inhibited the LPS-induced increase in CD11b mRNA (Fig. 1D).

**Figure 2:**
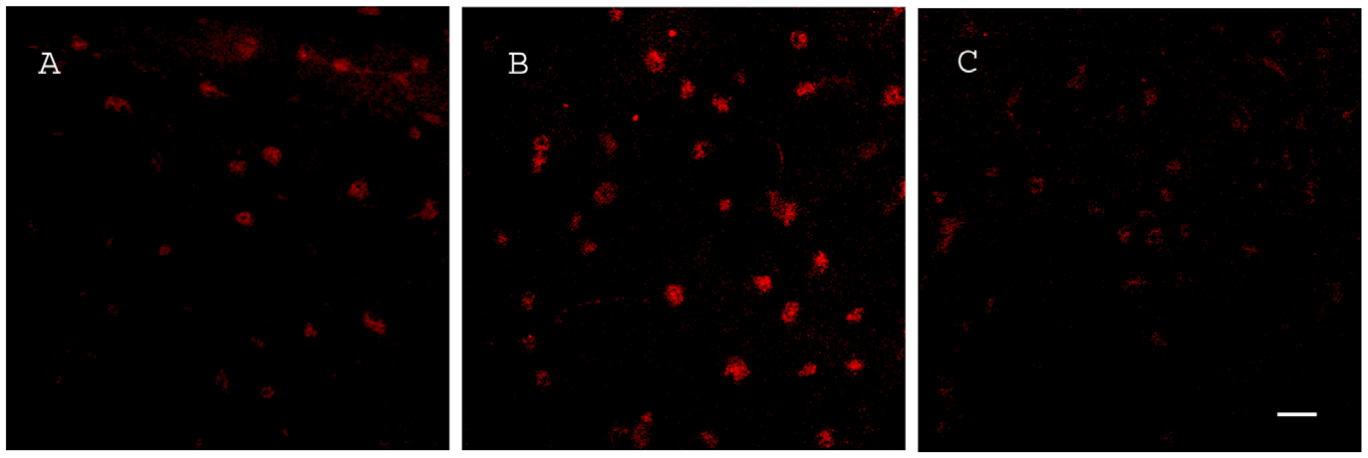
Effects of COL-3 on LPS-induced p38 MAPK activation in the brain. Confocal laser microscopy images showing immunoreactivity of p38 MAPK in the anterior commissure of fresh frozen sections of mice brains treated with vehicle (MC) (A), LPS (B) and LPS+40 mg/kg COL-3 (C) at 24 h post inoculation. Immunoreactivity of p38 MAPK was increased in LPS inoculated mice (B) compared to control mice (A) and was reduced by treatment with COL-3 (C). Scale bar: 20 µm.

**Figure 3:**
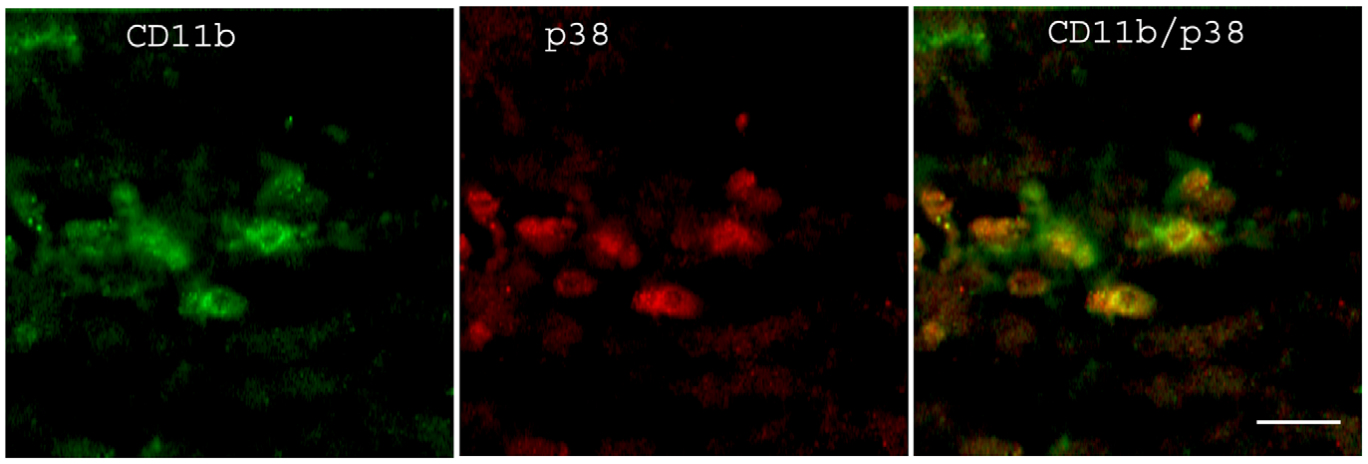
Expression of p38 MAPK within LPS-activated microglial cells. Double labeling immunofluorescence confocal laser microscopy images showing the co-localization of CD11b (green) and p38 MAPK (red) in the corpus callosum of mice brains at 24 h post inoculation inoculated with LPS. Scale bar: 20 µm.

LPS-inoculated animals showed high reactivity of p38 MAPK compared to vehicle-injected controls. Treatment with COL-3 reduced the LPS-induced increase in p38 MAPK immunoreactivity (Fig. 2). Double immunoflourescence showed co-localization of CD11b and p38 MAPK staining in LPS treated animals, indicating up-regulation of p38 MAPK within microglial cells (Fig. 3).

### 3.2. Effect of COL-3 on LPS-induced Changes in Proinflammatory Cytokine Expression

**Figure 4:**
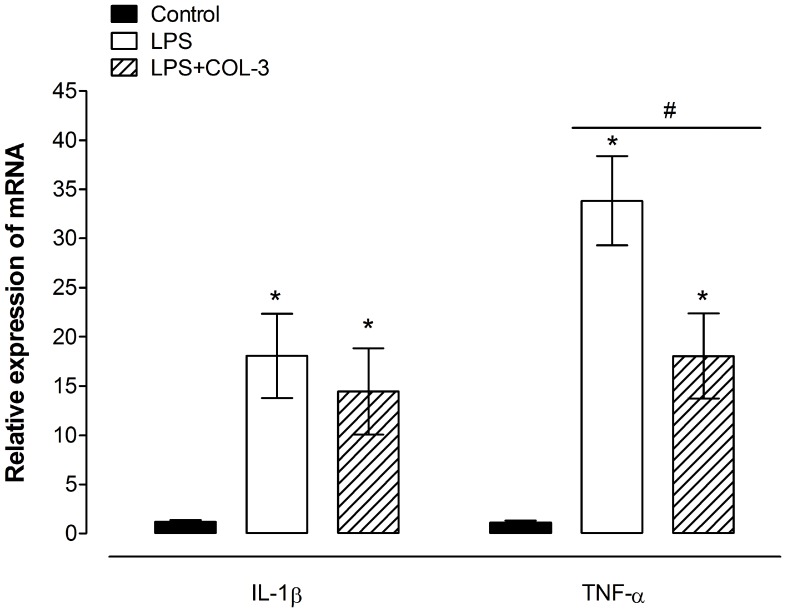
Effects of COL-3 on LPS-induced up-regulation of cytokine transcript levels in the brain. Relative expression of IL-1β and TNF-α mRNA in brains of control mice, LPS-inoculated vehicle-treated and LPS-inoculated COL-3-treated mice at 24 h post LPS inoculation. Each bar represents the mean ± S.E.M of the values obtained from 4 (for TNF-α) –12 (for IL-1β) animals. *P<0.05 compared to control animals and #P<0.05 compared to LPS-inoculated mice pretreated with vehicle.

The levels of the cytokines IL-1β and TNF-α mRNA were increased in the brains of LPS-inoculated mice compared to vehicle-injected controls. Treatment with COL-3 significantly reduced LPS-induced TNF-α, but not IL-1β mRNA expression (Fig. 4).

### 3.3. Antibacterial Activity of COL-3

COL-3 had no bacterial activity against a Gram-negative bacteria, *Escherichia coli*, but had antibacterial activity against a Gram-positive bacteria *Staphylococcus aureus*, with a MIC of 1 mg/ml.

## Discussion

The results of this study show for the first time that the chemically modified tetracycline COL-3, which has been described to lack antibacterial activity, has antibacterial activity against *S. aureus* but not *E. coli*, inhibits both the LPS-induced microglia activation and LPS -induced elevation in the transcripts of the cytokine TNF-α.

COL-3 has been described and cited to be a CMT which lack antibacterial activity by many research groups and has also been included in various reviews as such [11], [35], [36]. Administration of CMT-1 for three weeks did not induce drug resistance in *E. coli* to tetracycline, whereas administration of the parent compound tetracycline for three weeks resulted in emergence of tetracycline-resistant *E. coli* [11]. Our studies showed that COL-3 had no antibacterial activity against *E. coli*, thus, might not be expected to induce drug resistance in this bacteria after chronic treatment, but could possibly induce resistance to *S. aureus* because it had antibacterial activity against this bacteria.

Activated microglia increase the expression of CD11b both at transcript and protein levels [37], [38], [39]. CD11b is part of the high molecular weight cell surface heterodimeric glycoprotein Mac-1(CD11b/CD18) and its expression in the brain is restricted to microglia [40]. It is considered as the microglia surface marker that has the most important functional significance [38]. The increase in CD11b has been reported to correlate with the intensity of microglial activation [41], [38] and it is therefore considered a useful indicator of microglia activation [40]. We observed increased CD11b at both transcript and protein level in the brain after intraperitoneal injection of LPS. Our findings are in agreement with previous studies that report that peripheral administration of LPS activates microglia [42], [23], which can be chronic and result in neurodegeneration [42].

The activation of the serine/threonine MAPK pathway links LPS-induced activation of immune cells to increased transcription and production of cytokines such as IL-1β and TNF-α [43], [44]. In agreement with previous studies we observed increased p38 MAPK immunoreactivity as well as increased levels of the transcripts of the cytokines TNF-α and IL-1β in the brain after administration of LPS [45], [42]. The p38 MAPK immunoreactivity was co-localized with CD11b immunoreactivity, suggesting an activation of p38 MAPK in microglia cells [46], [47], [48], [49], which could have eventually led to increased cytokine production.

Previous studies have shown that minocycline, a semi-synthetic tetracycline antibiotic, inhibits LPS-induced microglia activation and cytokine production in the brain [25], [22]. COL-3 has also been reported to inhibit pro-inflammatory functions of whole blood cells or specific immune cells, such as mast cells, *in vitro* including inhibition of activation-induced mRNA expression and secretion of cytokines such as IL-1β, IL-6, IL-8 and TNF-α [50], [51]. Surprisingly, we observed that COL-3 inhibited LPS-induced increase in transcripts of TNF-α, but not IL-1β, in the brain whilst minocycline inhibits the transcript levels of both cytokines. We still do not know the exact reason of this selective inhibition. This could be a kinetic issue since minocycline has been reported to inhibit manganese-induced increase in IL-1β in rats on days 1 and 7, whilst it inhibited the manganese-induced increase in TNF-α only on day 7 post manganese injection [52]. In addition, the differential effects on the production of these two cytokines has also been observed with another tetracycline antibiotic, doxycycline, which was reported to reduce the production of IL-1β but not TNF-α in activated microglia when one time point was measured [53].

Minocycline’s inhibitory effects on LPS-induced microglia activation and cytokine production in the brain is possibly via inhibition of p38 MAPK activation [54], [55], [56]. Treatment with COL-3 inhibited both LPS-induced increase in CD11b and p38 MAPK immunoreactivity as well as the transcript level of TNF-α. These results suggest that COL-3 inhibits LPS-induced microglia activation and cytokine production, possibly via inhibition of the p38 MAPK pathway in a manner similar to minocycline. COL-3 inhibited calcium ionophore-induced protein kinase C (PKC) activity in mast cells [50], which could have contributed to inhibition of mast cell activation and cytokine production. PKC isoforms have been found to activate the MAPK pathway [57]. COL-3 and other CMTs have been proposed to produce their activities as MMP inhibitors by binding to metal ions including calcium [35]. COL-3 has also been reported to reduce the production of TNF-alpha in mast cells activated by phorbol-12-myristate-13-acetate (PMA) plus a calcium ionophore [58], [50]. Thus, it is plausible that COL-3 could inhibit p38 MAPK via binding to calcium and/or inhibition of PKC, eventually inhibiting cytokine production by microglia. The effects of COL-3 on LPS-induced activation of the microglia serine/threonine MAPK pathway and cytokine production warrants further research using cell culture and *in vitro* models. Interestingly, the dose of COL-3, (40 mg/kg) which we found to be effective for preventing LPS-induced inflammatory activities in the CNS is very similar to the clinically effective dose (50 mg/kg) used for treatment of sarcomas [15], [14].

Minocycline has been found to have neuroprotective effects in animal models of neurodegenerative diseases and has been evaluated in clinical trials for a number of neurodegenerative or autoimmune diseases which affect the CNS including Parkinson’s disease, Huntington’s disease and multiple sclerosis. These neuroprotective activities of minocycline have been attributed to its inhibitory effects on microglia activation [6], [7], [59], [8], [9], [10]. Taking into consideration our current findings, the neuroprotective effects of COL-3 warrant further research as a potential candidate to manage conditions where neuroinflammation plays a pathogenic role. COL-3 might have a slight advantage over minocycline in that it possibly has a limited antibacterial spectrum of activity compared to minocycline, since it had no activity against *E.coli* whereas minocycline has, thus treatment with COL-3 for long periods would not induce widespread bacterial resistance to tetracycline antibiotics, a problem likely to occur with minocycline.
